# Wild birds in Chile Harbor diverse avian influenza A viruses

**DOI:** 10.1038/s41426-018-0046-9

**Published:** 2018-03-29

**Authors:** Pedro Jiménez-Bluhm, Erik A. Karlsson, Pamela Freiden, Bridgett Sharp, Francisca Di Pillo, Jorge E. Osorio, Christopher Hamilton-West, Stacey Schultz-Cherry

**Affiliations:** 1 Epidemiology Unit, Department of Preventative Veterinary Medicine, Faculty of Veterinary Sciences, University of Chile, Santiago, Chile; 2 Institut Pasteur-Cambodia, Phnom Penh, Cambodia; 3 St Jude Children’s Research Hospital, Memphis, TN 38105 USA; 4 St Jude Children’s Research Hospital, Memphis, TN 38105 USA; 5 Epidemiology Unit, Department of Preventative Veterinary Medicine, Faculty of Veterinary Sciences, University of Chile, Santiago, Chile; 6 University of Wisconsin-Madison, Madison, WI 53706 USA; 7 Epidemiology Unit, Department of Preventative Veterinary Medicine, Faculty of Veterinary Sciences, University of Chile, Santiago, Chile; 8 St Jude Children’s Research Hospital, Memphis, TN 38105 USA

## Abstract

While the circulation of avian influenza viruses (IAV) in wild birds in the northern hemisphere has been well documented, data from South America remain sparse. To address this gap in knowledge, we undertook IAV surveillance in wild birds in parts of Central and Northern Chile between 2012 and 2015. A wide diversity of hemagglutinin (HA) and neuraminidase (NA) subtypes were identified and 16 viruses were isolated including low pathogenic H5 and H7 strains, making this the largest and most diverse collection of Chilean avian IAVs to date. Unlike IAVs isolated from wild birds in other South American countries where the genes were most like viruses isolated from wild birds in either North America or South America, the Chilean viruses were reassortants containing genes like viruses isolated from both continents. In summary, our studies demonstrate that genetically diverse avian IAVs are circulating in wild birds in Chile highlighting the need for further investigation in this understudied area of the world.

## Introduction

While much is known about avian influenza A virus (IAV) prevalence in Eurasian and North American wild birds^1–3^, widespread surveillance is lacking in much of the Southern Hemisphere ^4^.

With over 4000 km of coast and hundreds of wetlands for wintering and breeding^5^, the Chilean mainland is home to many migrant species including those on the Pacific and Atlantic flyways^2, 4^. Yet little is known about IAV in Chilean bird populations. Previous reports are limited to the isolation of three low pathogenic avian influenza (LPAI) subtypes most closely related to viruses isolated from North American shorebirds, an H4N8 virus in domestic turkey, and a highly pathogenic avian influenza (HPAI) H7N3 virus in commercial poultry in 2002^5–7^. Most recently, a LPAI H7N6 virus was detected in a commercial turkey farm in central Chile that also had origins in wild birds (H.-W., in preparation). Thus, the goal of this study was to begin defining the prevalence and diversity of AIVs in Chilean wild birds.

## Materials and methods

### Sample sites and sample collection

**Fig. 1:**
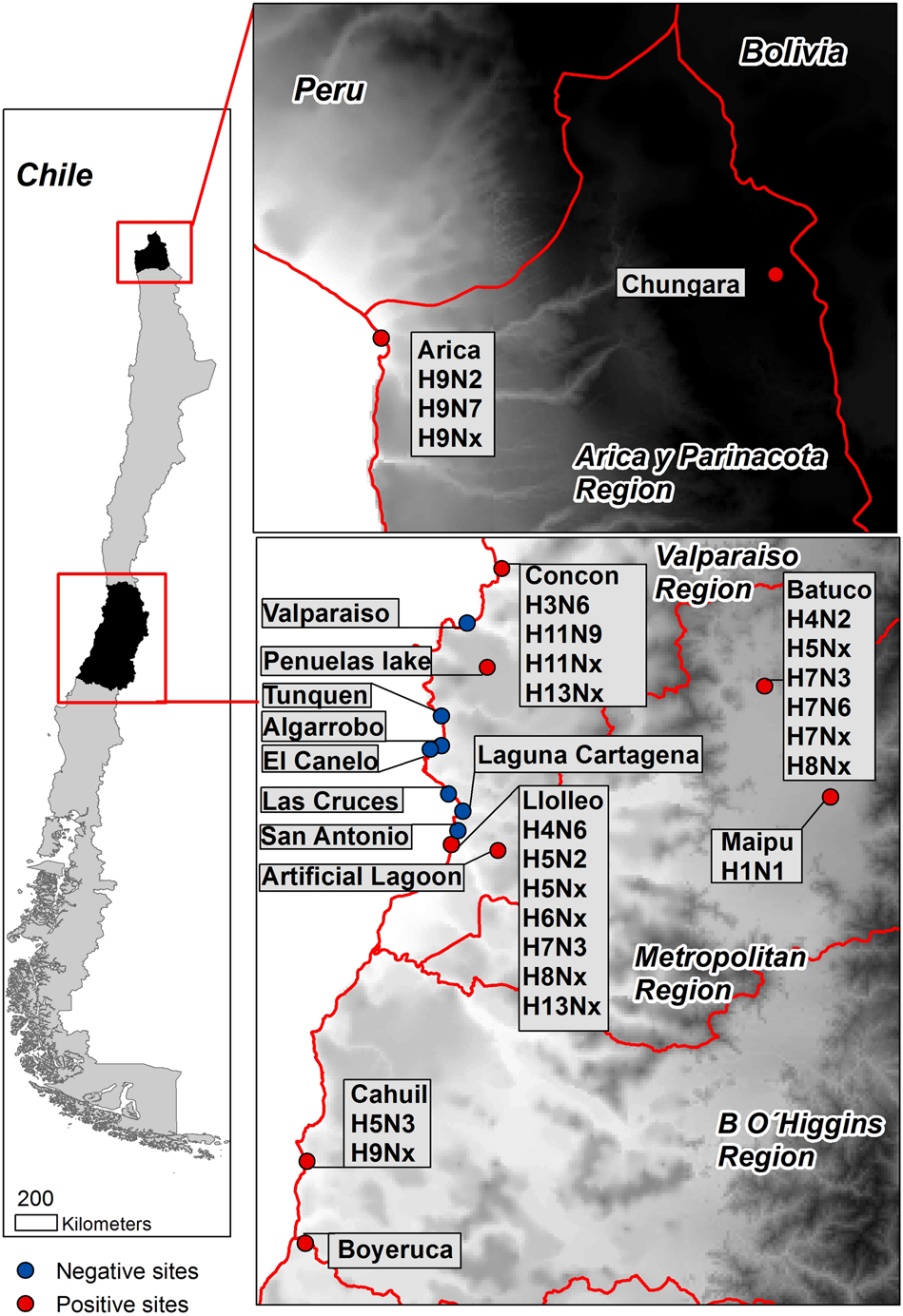
Location of surveillance sites in Chile. Positive sites are in red while negative sites are in blue. Subtypes obtained at each site are indicated in callout boxes

From June 2012 to September 2015, 4036 fresh wild bird feces were collected from 23 sampling sites consisting of wetlands, shorelines, estuaries, and lagoons in Central and Northern Chile (Fig. 1) as described^8^. Given the lack of data regarding IAV in Chilean wild birds, we conducted exploratory seasonal sampling in June to July 2012 (*n* = 216), March 2013 (*n* = 379), November 2013 (*n* = 899), and April 2014 (*n* = 1138) before undertaking a more targeted, risk-based approach to identify the optimal sites and sample numbers to ensure statistical power.

#### Surveillance site selection

All sites recognized as wild bird concentration areas in Chile were characterized by species diversity, number of inter hemispheric migratory species, number of resident species, and species already recognized as reservoirs of IAV. For each variable four categories were created (by the median and Q1 and Q3). Then each variable was weighted by researcher’s knowledge (10%, 10%, 10%, and 70%, respectively). With this information, a risk score was calculated for each site to focus the surveillance in high-risk areas for IAV.

#### Calculating statistical power

The wild bird concentration at the sites was considered our sampling unit. Our target population was the feces at each site, and we assumed that from each site we would have at least 1000 fresh feces. Based on the following formula^9^ to allow the identification of at least one positive sample, assuming a prevalence of 1.3%, and a confidence level of 95%, 205 samples must be collected.

$$n = \left( 1 \right. - \left( \propto \right) \wedge \left( {1{\mathrm{/}}D} \right) \ast \left( {N - \left( {D - 1} \right){\mathrm{/}}2} \right),$$

where

- *n* = required sampling size
- ∝ = 1-confidence level
- *D* = estimated minimum number of positive samples (population size * expected prevalence)
- *N* = population size

### Sample sc**r**eening and virus isolation

Viral RNA was extracted from 50 μl of the swab sample on a Kingfisher Flex Magnetic Particle Processor (Thermo Fisher Scientific, USA) using the Ambion MagMAX-96 AI/ND Viral RNA Isolation kit (Life Technologies Corporation, Grand Island, NY, USA)^10^. RNA was screened using a Bio-Rad CFX96 Real-Time PCR Detection System on a C1000 Thermocycler (Bio-Rad, Hercules, CA, USA), with TaqMan Fast Virus 1-Step Master Mix (Applied Biosystems, Foster City, CA, USA) and primers/probe specific for the influenza M gene^11^. Samples with a fluorescence cycle threshold (Ct) value ≤38 were considered positive. Virus isolation was attempted in embryonated chicken eggs on all samples with a Ct ≤35 as previously described^12^. Host species were identified using primers designed to amplify a segment of the mitochondrial cytochrome-oxidase I as described ^13^.

### Virus sequencing

Full-length genomes were obtained using universal oligonucleotide primer sets^14^ and Sanger sequencing as previously described^8^. Sequences can be accessed under GenBank numbers KX101128 to KX101209 and KX185892 to KX185931.

### Phylogenetic analysis

Influenza gene sequences were obtained from the NCBI Influenza Virus Database (https://www.ncbi.nlm.nih.gov/genomes/FLU/Database) as accessed in November 2017. Only full-length genes from the Americas, Europe, and Asian avian strains were included while duplicates were excluded. Sequence assembly, visual inspection, and trimming to remove nucleotides outside the coding region was performed using BioEdit version 7.2.5 (ref. ^15^) and alignment performed with MUSCLE version 3.8.3 (ref. ^16^). The best-fit nucleotide substitution models were selected individually for each gene by ModelTest in in Mega 7 (ref. ^17^). Phylogenic relationships for each gene were inferred by Maximum Likelihood (ML), incorporating a general time-reversible model of nucleotide substitution with a gamma-distributed rate variation among sites and a proportion of invariant sites (GTR + G + I). One thousand bootstrap replicates were performed to infer the robustness of the ML trees using RaxML version 8.0 (ref. ^18^). ML inference was repeated at least three times per dataset to assure tree topology was maintained. Final trees were constructed using TreeGraph2 and FigTree v1.4.3 (ref. ^19^). For the analysis of internal genes, we obtained all publicly available genes of wild bird origin obtained between 1976 and 2017 from Eurasia and the Americas. We then randomly selected 10 strains/year/location per gene for the analysis. This process was repeated at least twice and final tees were run at least three times to assure consistency in consultation with Dr. Justin Bahl. The final number of selected taxa per tree is specified in Supplemental Figures S1 to S22 that include expanded trees showing all sequences included for analysis.

### Statistical analysis

All statistics were calculated using Excel 2013 (Microsoft Corporation, Redmond, WA, USA). Graphs were produced using GraphPad Prism software (La Jolla, CA, USA).

## Results

### AIV prevalence and host species

Of the 4036 fecal samples collected, 115 were positive for influenza virus M gene by rRT-PCR. Prevalence differed by season (Table S1) and site (Table S2). Comparisons between seasons (winter/spring) v/s (summer/fall) found that summer/fall had a higher positivity than winter/spring (Wilcoxon test, *p* = 0.007). Not surprisingly, the three most intensely sampled sites accounted for over half the total sampling effort (*n* = 2508) and yielded 73% of the overall positives samples. Twelve bird species, primarily from the *Anseriformes* order including Yellow-billed pintails (*Anas georgica*) and Yellow-billed teals (*Anas flavrostris*), were identified as the primary host (Table S3). They also supported the largest strain diversity. Other host species included Chiloé wigeon (*Anas sibilatrix*), mallards (*Anas platyrhynchos*), Red-fronted coot (*Fulica rufifrons*), oystercatchers (*Haematopus*), gulls (*Larus*), Black necked stilt (*Himantopus mexicanus*), Gray plover (*Pluvialis squatarola*), and Whimbrel (*Numenius phaeopus*) (Table S2).

### Viral diversity and unique reassortants

Full genomic sequences were obtained from the 16 isolates and partial sequences were obtained from 20 positive swab samples. Diverse HA (H1, H3, H4, H5, H6, H7, H8, H9, H11, and H13) and NA (N1, N2, N3, N6, and N9) subtypes were identified for a total of 11 HA/NA combination (Fig. 1 and Table S2). While similar IAV subtype diversity was described in samples collected between 2006 and 2011 in Perú^20^, the Chilean viruses contain unique combinations of genes like viruses isolated from both North and South America (Table S4). The most diverse viruses identified were the A/American oystercatcher/Chile/C1307/2015 and A/Grey Plover/Chile/C1313/2015 H9 viruses isolated from Northern Chile (Fig. 1 and Table S4), which are 4 + 4 and 6 + 2 North to South American lineage viruses, respectively. The phylogenetic trees for each virus and gene can be found in Supplementary Figures S1 to S21. These data highlight that influenza viruses in Chilean wild birds harbor widespread HA and NA diversity with unique combinations of genes found in both North and South America, unlike viruses from elsewhere in the American continents.

## Discussion

This heavy intermix of gene segments from different origins found in Chilean IAVs breaks with the paradigm of an isolated gene pool found in South America^20–22^. IAVs isolated from wild birds in Argentina were composed of genes unique to viruses isolated in South America^21, 23^, while Colombian wild bird viruses were most similar to those isolated in North America^8, 10^. In contrast, wild birds in Brazil had viruses from both lineages but not reassortants^24^. These data suggest that Chile may be a possible point of confluence where North and South American IAVs intermix, contrary to what has been reported in neighboring countries, like Argentina, Brazil, Peru, and Colombia^8, 10, 20, 24–27^. Geographically and evolutionarily, it is intriguing to speculate that genetic diversity in Chilean wild birds could possibly be predicted by latitude. Wild birds in Northern Chile may have more frequent exposure to North American lineage viruses versus those in Central or Southern Chile; i.e. the further South you are in Chile, the less influx of North American gene segments and a greater presence of South American lineage genes. However, further surveillance in Chile as well as the rest of South America is necessary to fully test this hypothesis. Further, the identification of H5 and H7 subtypes is concerning given the risk to domestic poultry. Active and serological surveillance is underway to determine if domestic poultry in backyard production systems in areas surrounding our wild bird surveillance sites have been exposed to the identified IAVs.

In summary, we describe the presence of a wide array of IAV subtypes in Chilean wild birds with unique genetic diversity. Increased surveillance is needed to better understand the role of Chile in this genetic diversity between North and South America, the ecology and epidemiology of IAV in Chile, and to understand the risk of these viruses to domestic animal populations.

## Electronic supplementary material

Supplemental Table S1

Supplemental Table S2

Supplemental Table 3

Supplemental Table 4

Supplemental Figure S1

Supplemental Figure S2

Supplemental Figure S3

Supplemental Figure S4

Supplemental Figure S5

Supplemental Figure S6

Supplemental Figure S7

Supplemental Figure S8

Supplemental Figure S9

Supplemental Figure S10

Supplemental Figure S11

Supplemental Figure S12

Supplemental Figure S13

Supplemental Figure S14

Supplemental Figure S15

Supplemental Figure S16

Supplemental Figure S17

Supplemental Figure S18

Supplemental Figure S19

Supplemental Figure S20

Supplemental Figure S21

Supplemental Figure S22
