# Supplementary material for: Wild birds in Chile Harbor diverse avian influenza A viruses
Source: Emerg Microbes Infect. 2018 Mar 29;7:44. doi: 10.1038/s41426-018-0046-9 (PMC5874252; doi:10.1038/s41426-018-0046-9)
Supplement: Supplementary file 1 — Supplemental Table S1 [file 41426_2018_46_MOESM1_ESM.pdf]

Supplementary Table 1. IAV prevalence by season.

Year	Season	Sampled	Positive (Prevalence %)	CI 95 (%)	Isolated (%)
2012	Winter	216	1 (0.46)	0.0-1.4	1 (100)
2013	Summer	379	7 (1.85)	0.5-3.2	0 (0)
2013	Spring	899	23 (2.56)	1.5-3.7	7 (30.4)
2014	Fall	1138	49 (4.3)	3.1-5.5	3 (6.1)
2015	Spring	1404	35 (2.5)	1.7-3.3	5 (14.3)
