# Supplementary material for: Wild birds in Chile Harbor diverse avian influenza A viruses
Source: Emerg Microbes Infect. 2018 Mar 29;7:44. doi: 10.1038/s41426-018-0046-9 (PMC5874252; doi:10.1038/s41426-018-0046-9)
Supplement: Supplementary file 2 — Supplemental Table S2 [file 41426_2018_46_MOESM2_ESM.pdf]

Supplementary Table 2. IAV prevalence and subtypes obtained by sampling site.

Site (Latitude/Longitude)	Total samples	% Positives (# positive)	Isolates	Isolate Subtype (GB[a] accession number)	Partial sequences (GB accession)
**Arica** (18.4139/ 70.3250)	233	5.15% (12)	2	**H9N2** (KX185900-KX185931)<br>**H9N7** (KX185899-KX185928)	**H9Nx**; **H9Nx**
**Batuco** (33.1995/70.7900)	879	2.04% (18)	5	**H4N2** (KX185892-KX185926)<br>**H4N2** (KX185895-KX185923)<br>**H7N3** (KX101139-KX101208)<br>**H7N6** (KX101138-KX101206)<br>**H7N6 (**KX101138-KX101204)	**H5Nx**; **H7Nx**; **H8Nx**
**Boyeruca** (34.7006/72.0259)	100	4.0% (4)	0		
**Cahuil** (34.4793/72.0206)	120	3.33% (4)	1	**H5N3** (KX185904-KX185927)	**H9Nx**
**Chungará** (18.2296/69.1838)	353	0.28% (1)	0		
**Concón** (32.8829/71.4966)	984	2.94% (29)	3	**H3N6** (KX101132-KX101185)<br>**H11N9** (KX101130-KX101203)<br>**H11N9** (KX101129-KX101171)	**H11Nx**; **H11Nx**; **H13Nx**; **H13Nx**
**Llolleo** (33.6266/71.6326)	645	5.73% (37)	4	**H4N6** (KX101159-KX101196)<br>**H4N6** (KX101131-KX101202)<br>**H7N3** (KX101140-KX101201)<br>**H7N3** (KX101144-KX101209)	**H5Nx**; **H5Nx**;<br>**H5Nx** (KX101182)<br>**H5Nx** (KX101141)<br>**H5Nx (**KX101134)<br>**H5Nx** (KX101207)<br>**H5N2** (KX254928, KX101145)<br>**H6Nx** (KX101161)<br>**H8Nx** (KX101156)<br>**H13Nx** (KX101188)
**Maipú** (33.4985/70.6119)	153	1.3% (2)	1	**H1N1** (KX101143-KX101205)	
**Peñuelas** (33.1491/71.5359)	220	1.36% (3)	0		
**Other sites (n=8)**[b]	349	1.43% (5)	0		
**TOTAL**	4036	2.84% (115)	16		

[a]GenBank (GB) accession number; [b]Other sites include Algarrobo (33.3600/71.6600); El Canelo (33.3700/71.6900); Las Cruces (33.4900/71.6400); San Antonio (33.5892/71.6152); Tunquen (33.2800/71.6600); Valparaiso Artificial Lagoon (33.0300/71.500 and 33.6431/71.5070); and L. Cartagena (33.5368/71.6022)
