# Supplementary material for: Wild birds in Chile Harbor diverse avian influenza A viruses
Source: Emerg Microbes Infect. 2018 Mar 29;7:44. doi: 10.1038/s41426-018-0046-9 (PMC5874252; doi:10.1038/s41426-018-0046-9)
Supplement: Supplementary file 3 — Supplemental Table 3 [file 41426_2018_46_MOESM3_ESM.pdf]

Supplementary Table 3. Bird species and associated viral subtypes identified during active surveillance in Chile.

**Host species**	**Order**	**IAV Subtype obtained**
Chiloé wigeon (*Anas sibilatrix*)	*Anseriformes*	H5N2
Mallard (*Anas platyrhynchos*)	*Anseriformes*	H4N2, H5Nx
Yellow-billed pintail (*Anas georgica*)	*Anseriformes*	H1N1, H4N2, H4N6, H5N3, H7N3, H5Nx
Yellow-billed teal (*Anas flavirostris*)	*Anseriformes*	H1N1, H7N3, H7N6, H5Nx, H6Nx, H7Nx, H8Nx
Red-fronted coot (*Fulica rufifrons*)	*Gruiformes*	H3N6
American oystercatcher (*Haematopus palliatus*)	*Charadriiformes*	H9N2
Blackish oystercatcher (*Haematopus ate*r)	*Charadriiformes*	H9Nx
Black necked stilt (*Himantopus mexicanus*)	*Charadriiformes*	H11N9
Gray plover (*Pluvialis squatarola*)	*Charadriiformes*	H9N7
Franklin's gull (*Larus pipixcan*)	*Charadriiformes*	H13Nx
Kelp gull (*Larus dominicanus*)	*Charadriiformes*	H13Nx
Whimbrel (*Numenius phaeopus*)	*Charadriifromes*	H9Nx
