# Supplementary material for: Wild birds in Chile Harbor diverse avian influenza A viruses
Source: Emerg Microbes Infect. 2018 Mar 29;7:44. doi: 10.1038/s41426-018-0046-9 (PMC5874252; doi:10.1038/s41426-018-0046-9)
Supplement: Supplementary file 4 — Supplemental Table 4 [file 41426_2018_46_MOESM4_ESM.pdf]

Supplementary Table 4. Genetic diversity of Chilean avian influenza viruses. Boxes indicate gene segment position as resolved by Maximum Likelihood analysis. Red = North American avian; blue = South American avian; yellow= equine. Alleles for the NS gene indicated as “A” or “B”.

Virus	Subtype	**PB2**	**PB1**	**PA**	**HA**	**NP**	**NA**	**M**	**NS**
A/Yellow-billed pintail/Chile/1/2012	H1N1								B
A/Red-fronted coot/Chile/5/2013	H3N6								A
A/Yellow-billed pintail/Chile/6/2014	H4N6								A
A/Yellow-billed pintail/Chile/7/2014	H4N6								A
A/Yellow-billed teal/Chile/C918/2015	H4N2								A
A/Mallard/Chile/C948/2015	H4N2								A
A/Yellow-billed pintail/Chile/C1267/2015	H5N3								A
A/Yellow-billed teal/Chile/8/2013	H7N6								B
A/Yellow-billed teal/Chile/9/2013	H7N6								B
A/Yellow-billed pintail/Chile/10/2014	H7N3								A
A/Yellow-billed pintail/Chile/11/2014	H7N3								A
A/Yellow-billed teal/Chile/12/2014	H7N3								B
A/American oystercatcher/Chile/C1307/2015	H9N2								A
A/Grey plover/Chile/C1313/2015	H9N7								A
A/Black-necked stilt/1/2013	H11N9								A
A/Black-necked stilt/2/2013	H11N9								A
