# Supplementary material for: Wild birds in Chile Harbor diverse avian influenza A viruses
Source: Emerg Microbes Infect. 2018 Mar 29;7:44. doi: 10.1038/s41426-018-0046-9 (PMC5874252; doi:10.1038/s41426-018-0046-9)

Supplementary Figure S1. Phylogenetic analysis of the Chilean H5 (**A**), H7 (**B**), and H9 (**C**) HA genes. Trees were generated using maximum likelihood method and a general time reversible substitution model with a gamma distribution. Bootstrap values (n=1000) >70 are indicated. Scale bars indicate substitutions per site. Isolated strains indicated in black and italic. Eurasian strains, blue; North American strains, pink; and South American strains, dark pink. Chilean sequences are highlighted with a purple dot.

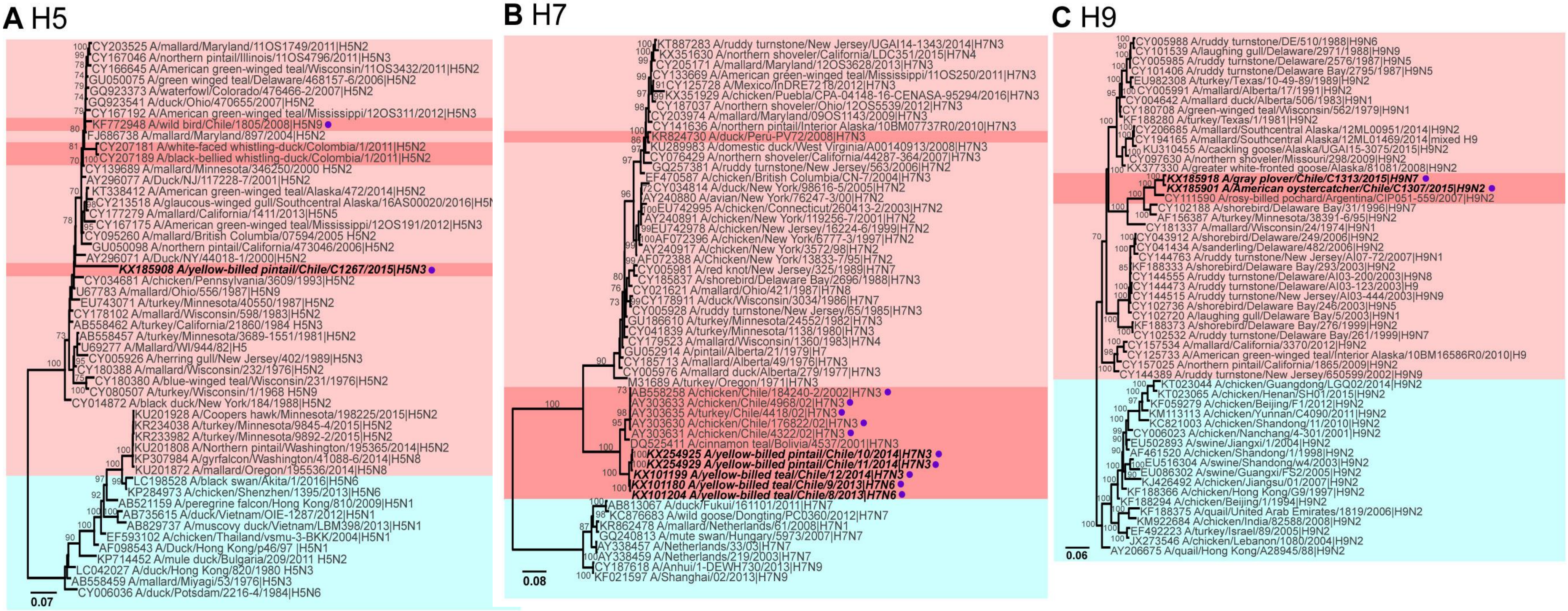

Supplement: Supplementary file 5 — Supplemental Figure S1 [file 41426_2018_46_MOESM5_ESM.pdf]
