# Supplementary material for: Wild birds in Chile Harbor diverse avian influenza A viruses
Source: Emerg Microbes Infect. 2018 Mar 29;7:44. doi: 10.1038/s41426-018-0046-9 (PMC5874252; doi:10.1038/s41426-018-0046-9)

Supplementary Figure S3 Phylogenetic tree of the matrix protein (MP) gene of wild bird viruses. Phylogenic analysis of complete MP genome sequences using maximum likelihood (RAxML) and incorporating a GTR+G+I substitution model with 1000 bootstrap replicates. Tree is midpoint rooted for clarity. Names and phylogenic position of the isolates obtained in this study indicated. 10 sequences/year/location when available were randomly selected from all data that was publicly available between 1976 and 2017. Tree was build out of North American (Red, n=414), Eurasian (Gray, n=238) and South American (Blue, n=65) wild bird sequences, including viruses collected in study. Global gull linage (purple) composed of a mix of origins. Major lineages indicated. Bootstrap support (≥70) for major nodes indicated. Scale bar indicates number of nucleotide substitutions per site.

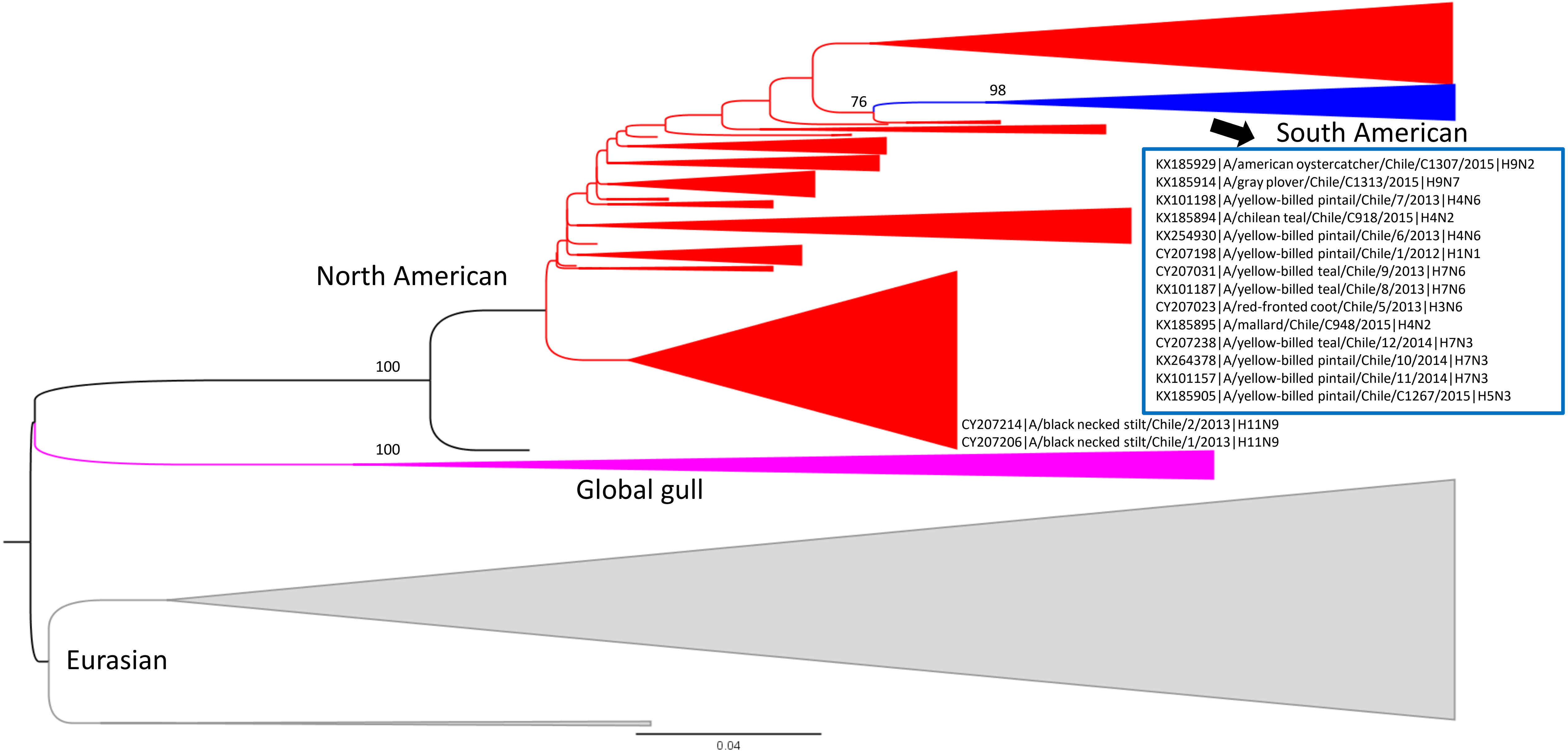

Supplement: Supplementary file 7 — Supplemental Figure S3 [file 41426_2018_46_MOESM7_ESM.pdf]
