# Supplementary material for: Wild birds in Chile Harbor diverse avian influenza A viruses
Source: Emerg Microbes Infect. 2018 Mar 29;7:44. doi: 10.1038/s41426-018-0046-9 (PMC5874252; doi:10.1038/s41426-018-0046-9)

Supplementary Figure S8 Phylogenetic tree of the H1 gene. Phylogenic analysis of complete H1 genome sequences using maximum likelihood (RAxML) and incorporating a GTR+G+I substitution model with 1000 bootstrap replicates. Names and phylogenic position of the isolates obtained in this study indicated in red. All bootstrap values shown. Tree is midpoint rooted for clarity. Clade colors: red, North American avian; blue, South American avian; light blue, Eurasian avian like swine; gray, Eurasian avian; green, pdmH1N1; brown, swine; purple, human. Scale bar indicates number of nucleotide substitutions per site.

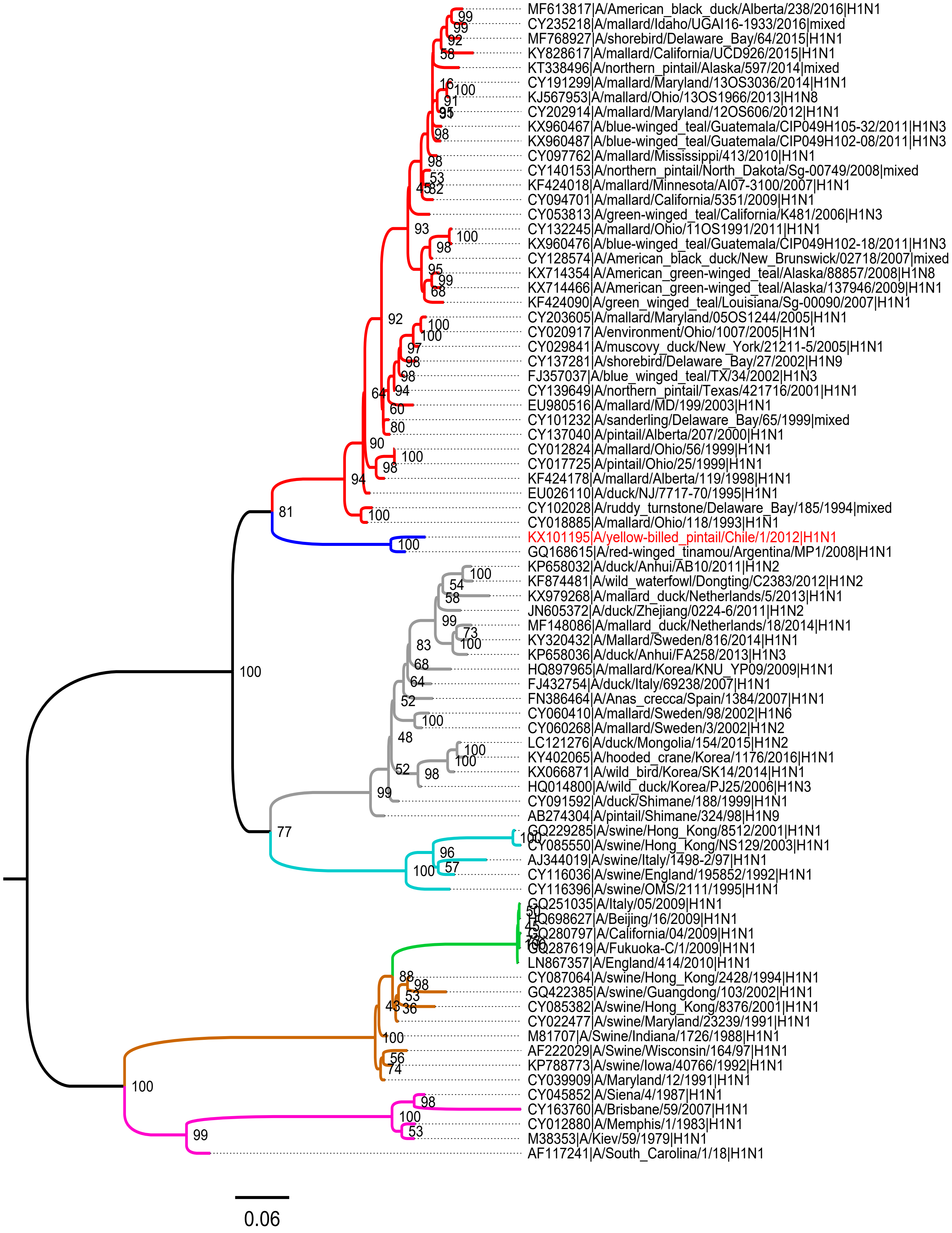

Supplement: Supplementary file 12 — Supplemental Figure S8 [file 41426_2018_46_MOESM12_ESM.pdf]
