# Supplementary material for: Wild birds in Chile Harbor diverse avian influenza A viruses
Source: Emerg Microbes Infect. 2018 Mar 29;7:44. doi: 10.1038/s41426-018-0046-9 (PMC5874252; doi:10.1038/s41426-018-0046-9)

Supplementary Figure S10 Phylogenetic tree of the H4 gene. Phylogenic analysis of complete H4 genome sequences using maximum likelihood (RAxML) and incorporating a GTR+G+I substitution model with 1000 bootstrap replicates. Names and phylogenic position of the isolates obtained in this study indicated in red. All bootstrap values shown. Tree is midpoint rooted for clarity. Clade colors as in figures S2-S7. Scale bar indicates number of nucleotide substitutions per site.

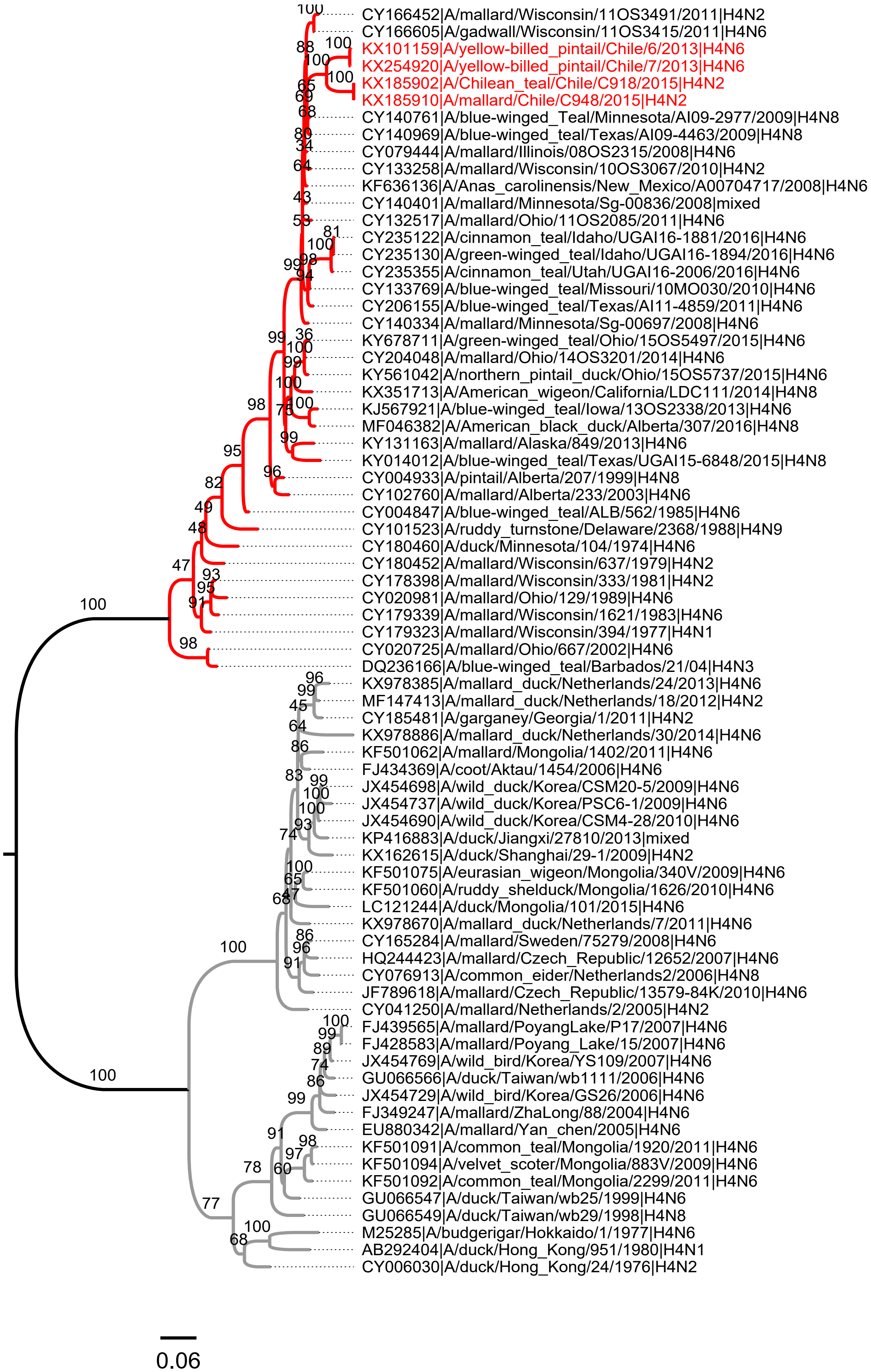

Supplement: Supplementary file 14 — Supplemental Figure S10 [file 41426_2018_46_MOESM14_ESM.pdf]
