# Supplementary material for: Wild birds in Chile Harbor diverse avian influenza A viruses
Source: Emerg Microbes Infect. 2018 Mar 29;7:44. doi: 10.1038/s41426-018-0046-9 (PMC5874252; doi:10.1038/s41426-018-0046-9)

Supplementary Figure S15 Phylogenetic tree of the N6 gene. Phylogenic analysis of complete N6 genome sequences using maximum likelihood (RAxML) and incorporating a GTR+G+I substitution model with 1000 bootstrap replicates. Names and phylogenic position of the isolates obtained in this study indicated in red. All bootstrap values shown. Tree is midpoint rooted for clarity. Clade colors as in figures S2-S7. Scale bar indicates number of nucleotide substitutions per site.

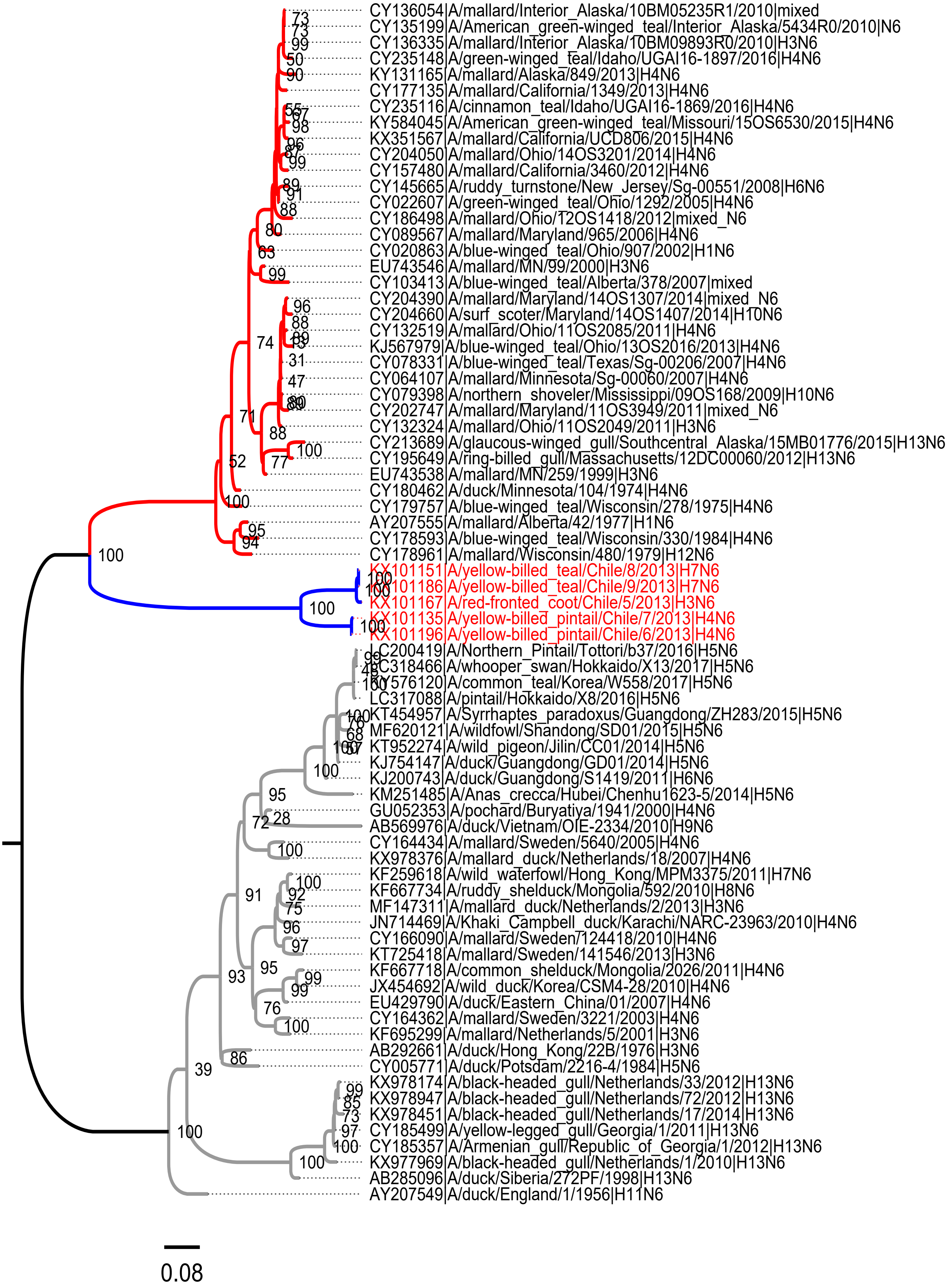

Supplement: Supplementary file 19 — Supplemental Figure S15 [file 41426_2018_46_MOESM19_ESM.pdf]
