# Supplementary material for: Wild birds in Chile Harbor diverse avian influenza A viruses
Source: Emerg Microbes Infect. 2018 Mar 29;7:44. doi: 10.1038/s41426-018-0046-9 (PMC5874252; doi:10.1038/s41426-018-0046-9)

Supplementary Figure S20 Expanded tree identical as supplementary Figure S5. Tip labels indicated. Sequences obtained in this study in red. All bootstrap values are shown. Tree is midpoint rooted for clarity. Clade colors as in figures S2-S7. Scale bar indicates number of nucleotide substitutions per site.

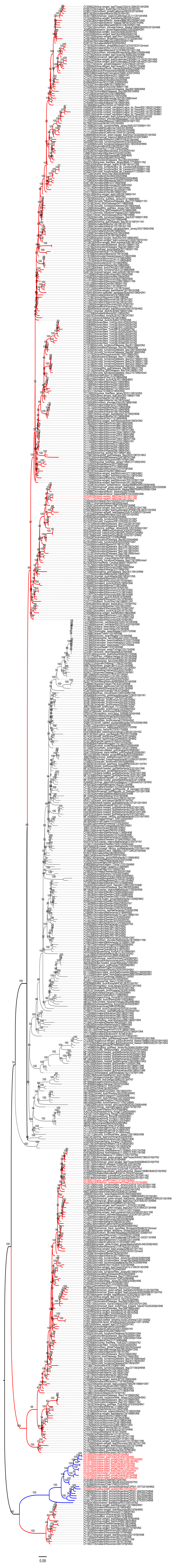

Supplement: Supplementary file 24 — Supplemental Figure S20 [file 41426_2018_46_MOESM24_ESM.pdf]
