# Supplementary material for: Wild birds in Chile Harbor diverse avian influenza A viruses
Source: Emerg Microbes Infect. 2018 Mar 29;7:44. doi: 10.1038/s41426-018-0046-9 (PMC5874252; doi:10.1038/s41426-018-0046-9)

**Supplementary Figure S9** Phylogenetic tree of the H3 gene. Phylogenic analysis of complete H3 genome sequences using maximum likelihood (RAxML) and incorporating a GTR+G+I substitution model with 1000 bootstrap replicates. **Figure S8 A**, Condensed tree; Equine (yellow), Avian North American (Red) and Avian Eurasian (Gray) lineages indicated. Isolate obtained in this study indicated with a red symbol. **Figure S8 B**, Expanded tree; same as S8 A. Names and phylogenic position of the isolate obtained in this study indicated in red. All publicly available full length avian H3 sequences (n=1594) of North American and Eurasian origin were selected, as well as mammalian non-human full length H3 sequences regardless of geographic origin (n=254). Tree is midpoint rooted for clarity. All bootstrap values shown. Scale bar indicates number of nucleotide substitutions per site

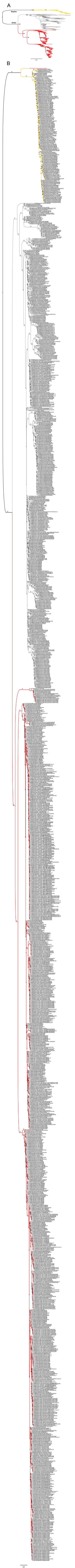

Supplement: Supplementary file 13 — Supplemental Figure S9 [file 41426_2018_46_MOESM13_ESM.pdf]
